# PREPARING FUTURE AGING PROFESSIONALS FOR A RACIALLY, INTERDISCIPLINARY, AND INTERGENERATIONALLY DIVERSE WORKFORCE

**DOI:** 10.1093/geroni/igz038.546

**Published:** 2019-11-08

**Authors:** Laura K Donorfio, Kathryn Elliott, Phyllis A Greenberg, Rona J Karasik, Kyoko Kishimoto

**Affiliations:** 1 Minnesota State University, Mankato, Minnesota, United States; 2 St. Cloud State University, St. Cloud, Minnesota, United States

## Abstract

Workforce development is a growing concern for educators and agencies serving older adults as both workers and aging clientele become more diverse (Obhi, et al., 2017; Spetz et al., 2015). Teaching courses on aging to traditionally-aged college students brings to light ageism, the continued need for cultural competence, and intergenerational experience. Typical pedagogical approaches of lecture and discussion only prepare future aging professionals for what Bloom (1956) calls the “cognitive domain”. Absent is the “affective domain” - how students interpret aging, the lifespan, and diversity in all its forms. For example, issues of power in the aging experience need to be analyzed to understand root causes of racial disparities, as well as the impact of past, current, and future policies. Preparing students for an intergenerational workforce is also essential, as there can be upwards to five generations working in paid positions together. With each generation comes differences in workplace expectations, modes of interacting (e.g., communicating, pace), and the impact of societal policies (e.g., institutional racism, ageism). Drawing upon the multiple disciplinary perspectives of its authors, this poster outlines the most prevalent differences among and between workforce generations, and identifies methods of pedagogy, andragogy, and geragogy needed to prepare future generations of aging professionals. Successful strategies and tools are highlighted including various experiential learning techniques (e.g., service-learning, simulations, vignettes, and anti-racist pedagogy).

